# In Which Patients Do the 2023 Duke-ISCVID Criteria for Infective Endocarditis Increase the Diagnosis of “Definite Endocarditis”?—A Preliminary Analysis in the Prospectively Evaluated DERIVE Cohort

**DOI:** 10.3390/jcm13164721

**Published:** 2024-08-12

**Authors:** Kirsten Schmidt-Hellerau, Johannes Camp, Philipp Alexander Marmulla, Siegbert Rieg, Norma Jung

**Affiliations:** 1 Department I of Internal Medicine, Faculty of Medicine and University Hospital Cologne, University of Cologne, 50937 Köln, Germany; pmarmull@smail.uni-koeln.de (P.A.M.); norma.jung@uk-koeln.de (N.J.); 2 Division of Infectious Diseases, Department of Medicine II, Medical Centre—University of Freiburg, Faculty of Medicine, University of Freiburg, 70196 Freiburg, Germany; johannes.camp@uniklinik-freiburg.de (J.C.); siegbert.rieg@uniklinik-freiburg.de (S.R.)

**Keywords:** endocarditis, modified Duke criteria, 2023 Duke-ISCVID criteria, diagnostic criteria

## Abstract

**Background/Objectives:** Recently, an update of the Duke criteria for the diagnosis of infectious endocarditis has been published: the 2023 Duke-ISCVID criteria. To gain an insight into which proportion of patients are affected by the new criteria, and which criteria might be the most relevant for the expected increase in sensitivity, we analysed data from a registry of cardiovascular infections. **Methods:** The 2023 Duke-ISCVID criteria were applied to patients who were diagnosed with and treated for endocarditis after having been classified as “possible” endocarditis according to the 2015 ESC Modified Duke criteria. In patients thus newly classified as “definite endocarditis”, diagnostic factors leading to this reclassification were described. **Results:** Of 397 patients, 48 (12%) did not fulfil the definition “definite infectious endocarditis” according to the 2015 ESC Modified Duke criteria. Of these, six (13%) fulfilled the definition when the 2023 Duke-ISCVID criteria were applied. A main factor triggering this reclassification was the consideration of microorganisms identified using valve PCR. **Conclusions:** As expected, the sensitivity of the new 2023 Duke-ISCVID criteria is increased in this cohort, mainly through the incorporation of new diagnostic methods in the criteria. Further studies are required to assess the effect on specificity in detail.

## 1. Introduction

Infective endocarditis (IE) is associated with high morbidity, and mortality remains around 30% [1]. Diagnosing IE is often challenging and diagnostic criteria are of great importance. Recommended in the guidelines of the European Society of Cardiology (ESC) and American Heart Association (AHA) are the Duke criteria [2,3]. The recently published 2023 Duke-International Society of Cardiovascular Diseases (ISCVID) Criteria [4] are an update of the 2000 Modified Duke criteria for infective endocarditis [5], aiming to adapt these diagnostic criteria to current changes in diagnostic methods and patient characteristics [4]. Already before this current update, the 2015 ESC guidelines adapted the 2000 Modified Duke criteria by considering certain findings demonstrated using cardiac computed tomography (CT) or via ^18^F-Fluorodeoxyglucose (FDG) positron emission tomography/computed tomography (PET/CT), or through single photon emission computed tomography (SPECT/CT) as a minor criterion.

The sensitivity of the new 2023 Duke-ISCVID criteria can be expected to increase, due to more extensive positive criteria and less strict requirements regarding blood culture sampling. The updates concern both pathological and clinical criteria. Among the changes are the consideration of polymerase chain reaction (PCR) findings, and of pathogens detected from materials such as leads of cardiovascular implantable electronic devices (CIED) or sterile body sites other than the heart. The definition of pathogens commonly causing IE has been refined, and formal requirements concerning the spacing and simultaneous drawing of blood cultures have been loosened. Also, a surgical major criterion has been added.

Aiming to estimate the magnitude of the effect of these updated criteria on the diagnostic classification of infectious endocarditis, we performed a preliminary analysis of data from the DERIVE registry (DEutsches RegIster kardioVaskulärer InfektionEn/German registry of cardiovascular infections). Apart from estimating the sensitivity of the 2023 Duke-ISCVID criteria compared to the 2015 ESC Modified Duke criteria, a further aim was to gain a detailed impression of which are the main clinical and diagnostic factors driving the change in classification from “possible IE” to “definite IE”, an information relevant for clinical practise and planning of further studies evaluating the 2023 Duke-ISCVID criteria.

## 2. Materials and Methods

The DERIVE registry is a multicentre prospective observational study of patients with cardiovascular infections in Germany; recruitment is currently still ongoing. In this registry, all patients can be included after a cardiovascular infection has been diagnosed (infective endocarditis, all infections associated with left ventricular assist devices (LVAD), infections of vascular grafts/endografts). Inclusion requires written informed consent (depending on the centre, patients not able to provide written consent themselves can be included if a legal guardian provides written consent). Patients under 18 years of age are excluded. Specific inclusion criteria for patients with IE are a classification of “possible IE” or “definite IE” by the 2015 ESC Modified Duke criteria. Data on patient characteristics, diagnostic findings, clinical course, complications and outcome are collected from the electronic hospital records and via follow up visits by phone at 90 days and 365 days. Pseudonymised data are registered into the password secured web application REDCap (https://www.project-redcap.org).

For the present substudy, we the extracted data of all patients with IE included at two of the sites (university hospital Freiburg and university hospital Cologne) from November 2019 to May 2023. Both sites have an active infectious disease consultation service and established interdisciplinary boards, enabling the identification of potential study participants as well as ensuring the quality of the case definition. We then analysed the data of patients in which IE had not been classified as “definite endocarditis” according to the 2015 ESC Modified Duke criteria, using the Statistical Package for the Social Sciences (SPSS) (version 28.0.1.1). Descriptive analysis included data on patient characteristics, characteristics of the infection and clinical and diagnostic information relevant for the Duke criteria. Absolute number and proportion were provided for categorical variables, whereas the median and interquartile ranges were provided for continuous variables. Following this, we applied the 2023 Duke-ISCVID criteria to patients in which the IE had not been classified as “definite endocarditis” according to the 2015 ESC Modified Duke criteria. In patients who were newly classified as “definite IE” according to the 2023 Duke-ISCVID criteria, the clinical factors leading to this change were described in detail.

## 3. Results

Of the 397 patients with IE, the 2015 ESC Modified Duke criteria classified 48 (12%) as “possible IE” (29/266 (11%) in one of the centres, 19/131 (15%) in the other). These patients were treated for a variety of pathogens, mainly oral streptococci, enterococci and staphylococci. In 16 (33%), the affected valve or IE associated findings could not be identified using echocardiography (see Table 1 for patient characteristics).

Of the 48 patients diagnosed with IE classified as “possible IE” by the 2015 ESC Modified Duke criteria, 6 (13%) were reclassified as “definite IE” applying the 2023 Duke-ISCVID criteria, corresponding to 2% of overall patients changing classification. The clinical factors leading to this reclassification were mainly the consideration of polymerase chain reaction (PCR) results in the pathologic and clinical criteria (positive valve PCR in four patients). Other relevant factors, in one patient each, were a positive culture from a sterile body site for a pathogen consistent with IE (new subitem of the clinical minor criterion “microbiologic evidence falling short of a major criterion”), and a positive culture of a CIED lead in the context of clinical signs of active IE (new pathologic criterion). In one of the patients, a further clinical criterion changed from minor to major: non-community-acquired Enterococcus faecalis had been identified in two blood cultures that had been drawn simultaneously. Please see Table 2 for details.

Setting the clinical diagnosis by experts and boards as the diagnostic standard, the sensitivity of the 2023 Duke-ISCVID criteria in this cohort was 89.4% (95% confidence interval (CI) 86.1–92.2%) compared to the 87.9% (CI 84.4–90.8) of the ESC 2015 Modified Duke criteria.

## 4. Discussion

In the setting of patients diagnosed with IE, the 2023 Duke-ISCVID criteria were found to have a higher sensitivity than the 2015 ESC Modified Duke criteria (89.4% vs. 87.9%) when compared to diagnosis by infectious disease specialists and interdisciplinary boards. The consideration of pathogen identification using PCR seems to be one of the main factors contributing to this increased sensitivity.

Of patients diagnosed with IE by infectious disease specialists and interdisciplinary boards (n = 397), 12% (n = 48) had not been classified as “definite IE”, but only as “possible IE” according to the 2015 ESC Modified Duke criteria. This proportion is within the expected range [6]. Applying the recently published 2023 Duke-ISCVID criteria to these patients, the classification changed to “definite IE” in 13% (6/48). An increase in sensitivity has also been described very recently in other cohorts of patients, depending on the study and the type of patients. Sensitivities ranged from 80.0% (among patients with certain blood stream infections) [7] up to 97.6% (among patients treated for IE) [6]. Several studies reported sensitivities in between these values: 81% in patients with *Staphylocococcus aureus* bacteraemia [8], and 84% among patients with suspected IE [9,10]. An absolute increase in sensitivity compared to the 2015 ESC Modified Duke criteria ranged from 2.6% to 4.2% [6,9,10].

New diagnostic methods had already been integrated in the 2015 ESC Modified Duke criteria in the form of CT and ^18^F-FDG PET/CT imaging. Of the current changes made to the diagnostic criteria, incorporating pathogens identified from explanted valves using PCR was responsible for most of the increase in sensitivity in the present study. In four of the six patients who were reclassified as definite IE, valve PCR was the factor (or among the factors) leading to this reclassification. When applying the 2015 Modified Duke criteria, major or even minor microbiological criteria had not been fulfilled.

Further factors that contributed to the increased sensitivity of the 2023 Duke-ISCVID criteria in the present study were loosened requirements regarding blood culture sampling (such as accepting, while explicitly not advising, blood cultures taken at the same site and deviations regarding timing of blood cultures), considering microbiological evidence from other materials (otherwise sterile body sites, CIED leads) and considering *E. faecalis* a typical pathogen independent from the place of acquisition. Changes due to other criteria such as the new surgical major criterion were not observed; in this case, this was due to the already positive major imaging criteria in all patients. Currently, PCR testing of explanted valves is not necessarily part of the routine management of patients with IE requiring surgery. While valve PCR testing is less likely to add relevant information in cases in which both the diagnosis of IE and the pathogen are already clearly established, our findings underline the important clinical value of valve PCR, especially in patients who do not yet fulfil the major microbiological criteria before the indicated valve surgery. It can not only confirm the diagnosis of IE when a detected pathogen could not yet be considered a major clinical criterion, but it can also establish diagnosis infections with organisms usually escaping blood and valve culture identification, such as a case of *Bartonella quintana* IE in this cohort.

In contrast to the 2015 ESC Modified Duke criteria, where predisposition was not clearly defined in detail, the 2023 Duke-ISCVID criteria lists clinical conditions that are defined as predispositions. In the six patients that changed classification, all predispositions that had been judged to be relevant when applying the 2000 Modified Duke criteria were found among the predispositions listed in the 2023 Duke-ISCVID criteria.

Due to the nature of the cohort (in which only patients with diagnosed IE are included), it is not possible to estimate the specificity of the 2023 Duke-ISCVID criteria. Meanwhile, very recent studies compared specificity to a previous version of the Duke criteria, reporting different estimations ranging from no significant loss of specificity [9] to a decrease from 74% to 60% [10]. A strength of the present study is the detailed analysis of which individual factors led to the fulfilment of diagnostic criteria that contributed to increased sensitivity. To our knowledge, this has not been investigated in similar detail yet. Apart from the clinical implications, our results provide information on which factors should be most urgently evaluated for their effect on specificity in order to further optimise the test performance of the Duke criteria in the future.

A further limitation of this study is that the inclusion of patients from two university hospitals might not be fully representative for all patients with IE; therefore, future multicentre studies involving lower levels of care are desirable. Nevertheless, an advantage of the tertiary care setting is the clinical experience with IE and the availability of specialist infectious disease, microbiology, cardiology and surgical consultation and interdisciplinary boards. This expertise is needed to ensure the quality of the clinical case definition that is required to determine the sensitivity of the diagnostic criteria. Another limitation of our study, reflecting the challenges of the real-world setting of clinical practise, is that of the four patients who were newly classified as having definite IE and had undergone valve surgery, no pathology report was available. Especially in the two patients that had only received 4 days of anti-infective treatment at the time of surgery, it might have been that pathological evidence would have resulted in positive 2015 ESC Modified Criteria.

## 5. Conclusions

The 2023 Duke-ISCVID criteria are of benefit, increasing diagnostic sensitivity in a subset of patients previously classified as “possible IE” by the 2015 ESC Modified Duke criteria. An expansion of the availability of the proposed novel (often PCR-based) diagnostic tools is important, as are further studies on the effect on the specificity of the respective tools.

## Figures and Tables

**Table 1:** Characteristics of patients diagnosed with infectious endocarditis (IE), classified as “possible IE” by the ESC 2015 Modified Duke criteria (n = 48).

Characteristics	N (%) or Median (IQR)
Age	69 (57.3–76.8)
Gender	
male	34 (71)
female	14 (29)
Charlson morbidity score	3 (2–4.75)
Mode of acquisition	
Community-acquired	24 (50)
Healthcare-associated	16 (33)
Nosocomial	8 (17)
Risk factors	
Prosthetic valve	16 (33)
Cardiac implantable electronic device	12 (25)
Left ventricular assist device	2 (4)
Other intracardiac material	5 (10)
Previous IE	3 (6)
Severe regurgitation	4 (8)
Intravenous drug use	1 (2)
Involved valve ^a^	
Aortic valve	17 (35)
Mitral valve	9 (19)
Pulmonary valve	2 (4)
Tricuspid valve	0 (0)
CIED	4 (8)
Echocardiography negative	16 (33)
Additional involved material	
LVAD	1 (2)
Vascular prosthesis	2 (4)
Extracardiac foci at presentation	
Osteoarticular	5 (10)
Visceral or deep seated abscess	3 (6)
Other ^b^	2 (4)
Complications	
Heart failure NYHA III-IV	6 (13)
Severe valvular regurgitation	9 (19)
Paravalvular abscess	3 (6)
Paravalvular fistula	3 (6)
Valve perforation	2 (4)
Stroke	6 (13)
Non-CNS embolisation ^c^	2 (4)
90 day mortality	8 (17)
Pathogens ^d^	
*Staphylococcus* spp.	12 (25)
*Staphylococcus aureus*	7 (15)
Coagulase-negative staphylococci ^e^	5 (10)
*Streptococcus* spp.	15 (31)
Oral streptococci	9 (19)
*Streptococcus* spp. other than oral or *S. pneumoniae*	6 (13)
*Enterococcus* spp.	5 (10)
*Enterococcus faecalis*	4 (8)
*Enterococcus faecium*	1 (2)
Others ^f^	10 (21)
Unknown	12 (25)

^a^ Multiple locations possible. ^b^ One each: endophthalmitis, brain abscess. ^c^ One each: lower extremity, kidney. ^d^ Polymicrobial infection (with 2 pathogens each) in 6 patients (13%). ^e^ Including n = 2 *Staphylococcus lugdunensis*. ^f^ One each: *Streptococcus pneumoniae*, *Serratia marcescens*, *Corynebacterium*, *Bacillus cereus*, *Bartonella quintana*, *Schaalia cardiffensis*, *Pseudomonas aeruginosa*, *Proteus mirabilis*, *Porphyromonas gingivalis*, *Candida tropicalis*. IE: infective endocarditis; CIED: cardiovascular implantable electronic device; LVAD: left ventricular assist device; NYHA: New York Heart Association Classification of Heart Failure; CNS: central nervous system.

**Table 2:** Characteristics of patients with IE classified as “possible IE” by the 2015 ESC Modified Duke criteria but as “definite IE” by the 2023 Duke-ISCVID criteria (n = 6).

	Patient 1	Patient 2	Patient 3	Patient 4	Patient 5	Patient 6
Age	70	62	55	57	79	46
Gender	Male	Male	Female	Male	Male	Male
Charlson comorbidity index	4	2	5	3	4	2
Mode of acquisition	Community-acquired	Healthcare-associated	Nosocomial	Community-acquired	Community-acquired	Community-acquired
Type of valve	Native	Native	Native	Native	Native	Native
Affected valve (imaging)	Mitral valve	Aortic valve	None (CIED lead vegetation)	Aortic valve	Aortic valve	Aortic and mitral valve
CIED	No	No	Pacemaker	No	No	No
Presenting symptoms	Pain and swelling of the left knee, dyspnoea	Hemi-hypaesthesia	Signs and symptoms of pacemaker pocket infection	Worsening dyspnoea	Nausea and worsening dyspnoea	Weight loss and sweats
2015 ESC Modified Duke criteria						
Major criteria	Imaging: MV mobile vegetation (9 × 5 mm)	Imaging: Valvular perforation	Imaging: Lead vegetation (10 × 8 mm)	Imaging: AV vegetation (10 × 10 mm)	Imaging: AV vegetation (16 × 9 mm)	Imaging: AV and MV vegetation (max. 13 mm)
Minor criteria	Predisposition: severe regurgitation, prosthetic material (MV) ^a^ Vascular: Lower extremity embolisation	Predisposition: persistent foramen ovale occluder ^a^ Pathogen: Not-community acquired *E. faecalis* in 2 blood cultures drawn at the same time	Predisposition: CIED	Predisposition: severe aortic valve regurgitation ^b^		Pathogen: one single blood culture positive for *S. anginosus*
2023 Duke-ISCVID criteria that differed from the 2000 Duke criteria						
Pathologic criteria		Explanted valve PCR positive for *E. faecalis*	Culture of extracted lead: *S. epidermidis*, identified in the context of clinical signs of active endocarditis	Explanted valve PCR positive for *Bartonella quintana*	Explanted valve PCR positive for *S. sanguinis*	Explanted valve PCR positive for *S. anginosus*
Clinical major criteria		Pathogen: Not-community acquired *E. faecalis* in 2 blood cultures drawn at the same time				
Clinical minor criteria	Positive culture for *S. sanguinis* from an intraoperative sample of the right knee					

^a^ “predisposition“ is not clearly defined in the 2015 ESC Modified Duke criteria; this type of implanted material can (as “previous valve repair” resp. “congenital anomaly with repair”) be classified as predisposition in the 2023 Duke-ISCVID criteria. ^b^ With recently developed symptoms, not classified as major criterion due to a lack of previous imaging. IE: infective endocarditis; CIED: cardiovascular implantable electronic device; MV: mitral valve; AV: aortic valve; mm: millimetre; PCR: polymerase chain reaction.

## Data Availability

Data available upon request.
